# Desialylation of platelet surface glycans enhances platelet adhesion to adsorbent polymers for lipoprotein apheresis

**DOI:** 10.1177/0391398820968849

**Published:** 2020-11-03

**Authors:** Lucia Lauková, René Weiss, Vladislav Semak, Viktoria Weber

**Affiliations:** 1 Department for Biomedical Research, Center for Biomedical Technology, Danube University Krems, Krems, Austria; 2 Department for Biomedical Research, Christian Doppler Laboratory for Innovative Therapy Approaches in Sepsis, Danube University Krems, Krems, Austria

**Keywords:** Platelets, sialylation, adsorption, glycosylation

## Abstract

**Background:**

Lipoprotein apheresis is an important therapeutic option in homozygous familial hypercholesterolemia, progressive atherosclerosis, or when depletion of lipoprotein(a) is indicated. It is generally regarded as safe, but drops in platelet counts as well as sporadic episodes of thrombocytopenia have been reported. We assessed the influence of platelet desialylation, which may be induced by endogenous or pathogen-derived neuraminidases, on platelet adhesion to polyacrylate-based adsorbents for whole blood lipoprotein apheresis.

**Methods:**

Medical grade platelet concentrates were incubated with neuraminidase in vitro and were circulated over adsorbent columns downscaled from clinical application.

**Results:**

Cleavage of terminal sialic residues resulted in platelet activation with significantly elevated expression of platelet factor 4 (PF4) and in enhanced platelet adhesion to the adsorbent, accompanied by a pronounced drop in platelet counts in the column flow-through.

**Conclusion:**

Alterations in endogenous neuraminidase activity or exogenous (pathogen-derived) neuraminidase may trigger enhanced platelet adhesion in whole blood lipoprotein apheresis.

## Introduction

The relation between elevated blood levels of low density lipoprotein cholesterol (LDL-C) and lipoprotein(a) [Lp(a)] and the progression of cardiovascular disease is well established.^1–3^

Individuals suffering from familial hypercholesterolemia (FH), an autosomal co-dominant disorder caused by mutations in the LDL receptor, are at particular risk of developing premature atherosclerotic cardiovascular disease (ASCVD) compared to normolipidemic individuals, as they often fail to adequately respond to lipid-lowering medications.^4–6^ In addition to LDL-C, Lp(a) is frequently elevated in FH, despite the lack of clear evidence of a crucial role of the LDL receptor in Lp(a) plasma clearance. Elevated Lp(a) concentrations are particularly deleterious for FH patients due to the pro-thrombotic and pro-inflammatory characteristics of Lp(a), and commonly do not respond well to lipid-lowering drugs.

Lipoprotein apheresis represents a therapeutic option for these indications of refractory dyslipidemia.^6–10^ Beyond lowering lipid levels, it exerts pleiotropic effects by influencing inflammatory parameters, such as cytokines, C-reactive protein, and oxidized phospholipids.^11–13^

While the majority of lipoprotein apheresis systems require plasma separation as first step, two apheresis systems are available for the direct adsorption of lipoproteins from whole blood using either polyacrylamide beads functionalized with acrylic acid (DALI) or cellulose beads functionalized with dextran sulfate (Liposorber D).^10^ Both systems efficiently deplete apolipoprotein B (apoB) containing lipoproteins by electrostatic interactions between positively charged apoB moieties and negatively charged polyacrylate or dextran sulfate ligands on the adsorbent surface. This direct contact of whole blood with the adsorbent demands a high degree of blood compatibility to minimize platelet activation and adhesion^14–16^ and to avoid triggering of coagulation.^17,18^ Thrombocytopenia has been occasionally reported even with clinically well-established hemoadsorption systems, requiring discontinuation of the treatment.^19,20^ While the underlying mechanisms remain unclear, alterations in platelet surface glycans, specifically a loss of terminal sialic acid (*N*-acetylneuraminic acid) residues, might be implicated in enhanced binding of platelets to the adsorbent polymers.^21–23^

Cooling of platelets induces irreversible clustering of glycan-bearing receptors on the platelet surface. Upon re-warming, platelets secrete endogenous neuraminidases that cleave terminal sialic acid residues from the platelet von Willebrand factor receptor, resulting in platelet clearance via hepatic receptors.^24^ In addition to this internal pool of platelet neuraminidases, pathogen-derived neuraminidases can induce alterations of platelet surface glycans during systemic infection. As an example, the pronounced thrombocytopenia associated with *Streptococcus pneumoniae* sepsis has been linked to receptor-dependent clearance of desialylated platelets rather than to the consumption of platelets in the course of septic disseminated intravascular coagulation.^25^

In the current study, we aimed to assess whether cleavage of terminal sialic acid residues of platelet surface glycans, for example, in the course of infection, resulting in a loss of negative platelet surface charge, may trigger enhanced platelet adhesion to adsorbent polymers in whole blood lipoprotein apheresis.

## Materials and methods

### Chemicals and reagents

Priming solution (134 mM Na+, 4 mM K+, 1.75 mM Ca2+, 0.5 mM Mg2+, 106.5 mM Cl−, 36 mM HCO3−) and acid citrate dextrose solution A (ACD-A; 22.0 g/l trisodium citrate dihydrate, 24.5 g/l glucose monohydrate, 7.3 g/l citric acid) were obtained from Fresenius Medical Care, Bad Homburg, Germany. Unfractionated heparin was purchased from Gilvasan Pharma, Vienna, Austria. Phosphate buffered saline (PBS) without Ca^2+^ and Mg^2+^ was from Life Technologies, Paisley, UK. Glutaraldehyde was obtained from Carl Roth, Karlsruhe, Germany.

### Human whole blood and platelet concentrates

Human whole blood was drawn from healthy volunteers into vacutainer tubes containing sodium citrate (Vacuette, Greiner Bio-One, Kremsmuenster, Austria), as approved by the Ethical Review Board of Danube University Krems. Medical grade platelet concentrates were obtained from the Clinic for Blood Group Serology and Transfusion Medicine, Medical University Vienna, Austria, as approved by the Ethics Committee (ECS2177/2015). They were produced using a Trima Accel^®^ automated blood collection system (Version 5.0, Gambro BCT, Lund, Sweden), stored in polyolefin bags in SSP^+^ solution (Macopharma, Tourcoing, France) at a ratio of 80% SSP^+^ and 20% plasma, and used within 2 days.

### Adsorbents

A hydrophilic adsorbent consisting of polyacrylamide beads functionalized with acrylic acid (DALI, Fresenius Medical Care, Bad Homburg, Germany) was used in this study. DALI, which is approved for the depletion of lipoproteins directly from whole blood, binds to the positively charged apoB100 containing lipoproteins LDL (low density lipoprotein) and lipoprotein(a) via electrostatic interactions.^20^ Adsorbent columns downscaled equivalent to clinical use (3.5 × 1.8 cm, adsorbent bed volume 8.9 ml) were packed with DALI beads, rinsed with 2 × 20 ml of priming solution containing ACD-A (1:40), and 10 IU/ml of heparin was supplemented during the first rinsing step, as recommended by the manufacturer.

### Neuraminidase treatment of platelets

The optimal conditions for cleavage of terminal sialic acid residues were determined in a series of pre-experiments. To this end, freshly drawn human whole blood was centrifuged (10 min, 500 g) at room temperature to isolate platelet rich plasma, which was re-centrifuged (10 min, 800 g) to obtain platelets. Platelets were resuspended in PBS to a final concentration of 3 × 10^8^/ml. Aliquots of 500 μl of the platelet suspension were incubated for 60 min at 37°C with 1, 2.5, 5, 10, and 20 mU neuraminidase from *Clostridium perfringens* (Roche, Mannheim, Germany) per 10^8^ platelets, or were left untreated. Platelets treated with 5 mU of neuraminidase were additionally analyzed at different time points (30, 60, 90, and 120 min). The presence of terminal sialic acid or galactose residues was analyzed by flow cytometry as described below.

### Recirculation of platelet concentrates over adsorbent columns

Platelet concentrates were diluted 1:3 in SSP^+^ medium and 1:2 in PBS to obtain a final platelet concentration of 3 × 10^8^/ml. Aliquots of 50 ml were incubated for 60 min at 37°C with neuraminidase (5 mU/10^8^ platelets), or left untreated. Thereafter, they were re-circulated over DALI columns (see above) for 4 h at a flow rate 1.2 ml/min using medical grade tubing sets (length: 170 cm, diameter: 3 mm, material: polyvinyl chloride containing di-2-ethylhexyl phthalate) and a hemodialysis roller pump (Fresenius Medical Care, Bad Homburg, Germany). Samples were drawn from the circuits immediately before starting the pump at the beginning of each experiment and after 1, 2, and 4 h of recirculation. Blood cells were quantified using a cell counter (Sysmex KX-21 N, Neumuenster, Germany).

### Scanning electron microscopy

Platelet adhesion to the adsorbent beads was assessed by scanning electron microscopy. After each recirculation experiment, the adsorbent cartridges were thoroughly rinsed with 100 ml of isotonic saline, and the adsorbent beads were removed from the cartridges and fixed in saline solution containing 2.5% glutaraldehyde. Samples were dehydrated using an ethanol gradient from 30% to 100%, dried for 12 h at room temperature, sputter-coated with gold (Q150R ES Sputter Coater, Quorum Technologies Ltd., East Sussex, UK), and analyzed with a FlexSEM 1000 scanning electron microscope (Hitachi, Tokyo, Japan).

### Flow cytometric characterization of platelets

The exposure of terminal sialic acid residues on platelet surface glycans was assessed by flow cytometry (CytoFLEX LX, Beckman Coulter, Brea, CA). Prior to analysis, all samples were diluted 1:100 in PBS. Sialic acid residues were detected using biotinylated *Maackia amurensis* lectin (MAL II, Vector Laboratories, Peterborough, UK), which is specific for terminal α-2,3-sialic acids, in combination with R-phycoerythrin (RPE)-conjugated streptavidin (Life Technologies). Terminal galactose residues were stained with biotinylated *Ricinus Communis* agglutinin (RCA, Vector Laboratories, Peterborough, UK) in combination with R-phycoerythrin (RPE)-conjugated streptavidin (Life Technologies, Paisley, UK). Platelet activation was analyzed with a phycoerythrin-cyanin (PC7)-conjugated anti-CD41 monoclonal antibody as platelet marker in combination with a fluorescein isothiocyanate (FITC)-conjugated anti-platelet factor 4 monoclonal antibody (anti-CXCL4, R&D Systems, Minneapolis, MN) or a fluorescein isothiocyanate (FITC)-conjugated anti-CD62p monoclonal antibody (Beckman Coulter, Brea, CA) as platelet activation markers. Data were acquired for 2 min at a flow rate of 10 µl/min and analyzed using the Kaluza Software 2.1 (Beckman Coulter, Brea, CA).

### Statistical analysis

Statistical analysis was performed using GraphPad Prism 8.0 (La Jolla, CA). One-way ANOVA and repeated measures one-way ANOVA followed by Tukey’s multiple comparisons test were used to analyze differences between neuraminidase concentrations and differences between incubation times. Repeated measures two-way ANOVA followed by Sidak’s multiple comparisons test was used to analyze differences between control and neuraminidase treated groups at different time points. Data are presented as means ± standard deviation (SD) of three or four independently performed experiments, and *p*-values ⩽0.05 were considered as statistically significant.

## Results

### Neuraminidase treatment to cleave terminal sialic acid residues

**Figure 1.**
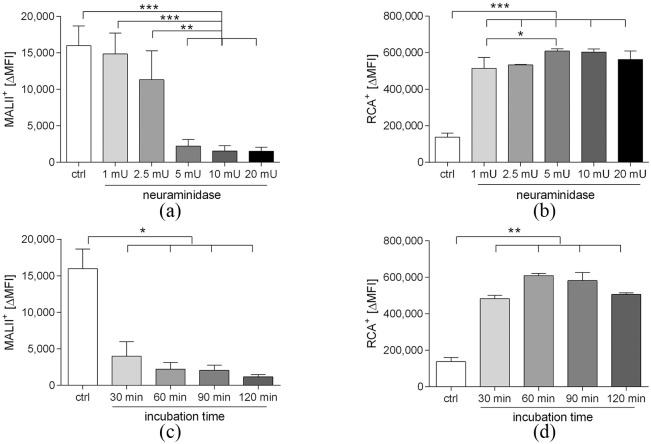
Desialylation of platelet glycoproteins. Platelets were incubated with neuraminidase as described in the Methods section to cleave terminal sialic acid residues. Exposure of sialic acid and galactose residues was analyzed by flow cytometry after staining with *Maackia amurensis* lectin (MALII; panels a and c) and *Ricinus Communis* agglutinin (RCA; panels b and d).*p < 0.05; **p < 0.01; ***p < 0.001; *n* = 3.

Treatment of platelets with neuraminidase resulted in a dose-dependent decrease of terminal sialic acid residues and, consequently, in an increase in terminal galactose residues. Doses higher than 5 mU neuraminidase per 10^8^ platelets did not result in further desialylation (Figure 1; *n* = 3). Incubation with 5 mU neuraminidase per 10^8^ platelets for 60 min was therefore used in all further experiments.

### Platelet desialylation enhances their adhesion to adsorbent polymers

**Figure 2.**
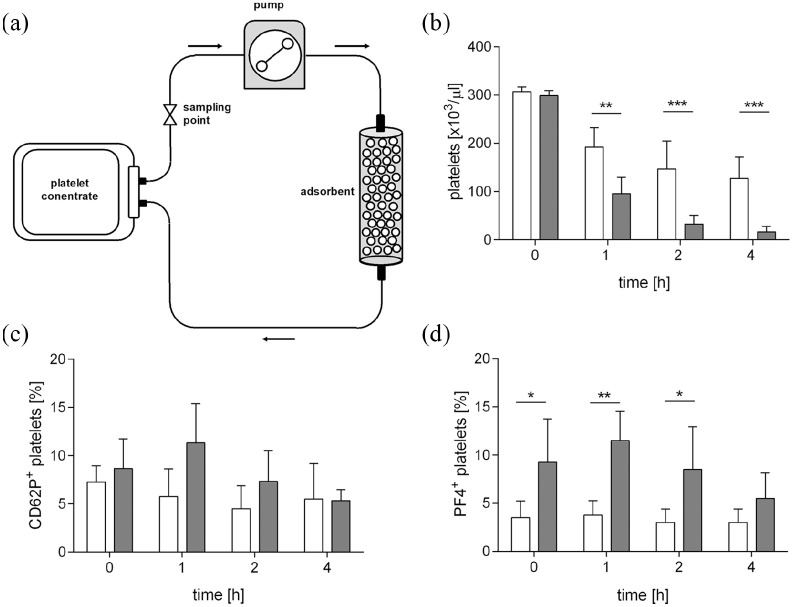
Recirculation of platelet concentrates over adsorbent columns. Platelets were treated with neuraminidase (filled bars) or were left untreated (open bars) and were recirculated over columns containing polyacrylate-based DALI beads as described in the Methods section (panel a). Platelet counts in the pool were quanitified by cell counting (panel b), and the percentage of activated platelets was assessed by flow cytometry using P-selectin (CD62p) and platelet factor 4 (PF4) surface expression as indicator for platelet activation (panels c-d). *p < 0.05; **p < 0.01; ***p < 0.001; *n* = 4.

**Figure 3.**
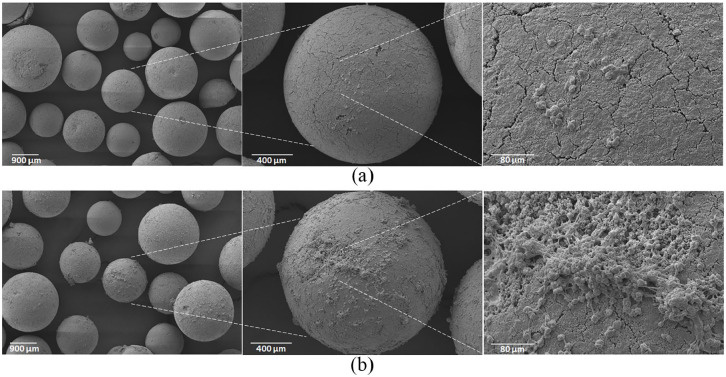
Scanning electron micrographs of adsorbent beads. Platelet concentrates without (panel a) and with (panel b) neuraminidase treatment were recirculated over DALI adsorbent columns as described in the Methods section. Thereafter, the columns were washed, fixed with glutaraldehyde, and analyzed using scanning electron microscopy.

Sialylation of platelets from platelet concentrates (Supplemental Figure S2(b), control bar) was comparable to sialylation of freshly isolated platelets from whole blood (Figure 1(a), control bar). Treatment of platelet concentrates with neuraminidase under the conditions described above cleaved about 62% of all terminal sialic acid residues (Supplemental Figure S2; *p* < 0.01, *n* = 4), and led to an increasing exposure of terminal galactose residues. Circulation of neuraminidase-treated platelet concentrates over adsorbent columns packed with DALI resulted in a significant decrease of platelet counts in the circulation over time (Figure 2), as compared to the untreated control (*p* < 0.001; *n* = 4). Neuraminidase treatment was associated with significantly increased expression of PF4, indicating platelet activation, while the increase in CD62p expression did not reach significance (Figure 2 and Supplemental Figure S1). The percentage of PF4^+^ platelets in the circulation decreased over time, most likely to the preferential binding of PF4^+^ platelets to the adsorbent polymer. This was confirmed by scanning electron microscopy, which revealed clusters of platelets adhering to the adsorbent surface for the neuraminidase-treated group, while there was only occasional adhesion of platelets to the polymer in the untreated control (Figure 3).

## Discussion

The exposure of whole blood to large adsorbent surfaces in therapeutic apheresis requires adsorbent polymers of particularly high blood compatibility to avoid adverse reactions at the blood-adsorbent interface,^26^ such as activation of immune cells or platelets. While device-related parameters including the chemical composition, charge, and morphology of adsorbent polymers and the choice of the anticoagulant can influence blood cell activation in the extracorporeal circuit,^18,27,28–30^ host-related factors may play a role, as well.

Most platelet membrane proteins, such as GPIb, GPIIb/IIIa, and GPIV, undergo post-translational modification and carry complex *N*- and *O*-linked carbohydrate chains, which are capped by sialic acid residues. Loss of these terminal sialic acids, which can be induced by endogenous or pathogen-derived neuraminiases, for example, in the context of autoimmune disease or infection,^22,23,31^ regulates the platelet life span by inducing platelet clearance via hepatic Ashwell Morrell receptors, and has been shown to affect platelet function, for example, by modulating thrombin-induced platelet activation.^32^

Here, we investigated whether desialylation of platelet surface glycans in vitro would result in enhanced platelet activation and in increased platelet adhesion to adsorbent polymers during DALI lipoprotein apheresis, which is generally well-tolerated and regarded as safe. A recent multicenter trial which prospectively evaluated 2154 DALI sessions confirmed low frequency of side effects, but reported drops in platelet counts in the range of 7% to 8%,^33^ and there are sporadic reports of severe thrombocytopenia in patients undergoing DALI treatment.^34^

In our study, we used medical grade thrombocyte concentrates instead of human whole blood to avoid clot formation during in vitro neuraminidase treatment; however, we adjusted the initial platelet concentration to that of whole blood. Incubation of platelet concentrates with *C. perfringens* neuraminidase, which preferentially targets α2,3-linked sialic acid residues, resulted in a dose-dependent cleavage of terminal sialic acid residues and in enhanced exposure of galactose residues, as shown by increased binding of RCA in flow cytometry. Desialylation was accompanied by platelet activation with increased surface expression of CD62 and PF4 in flow cytometry. Circulation of neuraminidase-treated platelet concentrates over miniaturized DALI columns led to enhanced platelet adhesion and strongly decreased platelet counts in the pool over time. The particular decrease of CD62^+^ and PF4^+^ platelets over time indicated the preferential adherence of activated platelets to the adsorbent cartridge. This implies that flow cytometry may actually have underestimated the degree of platelet activation, since activated platelets were “depleted” from the circuit. While soluble PF4 may appear superior for assessing the full extent of platelet activation in this context, its strong positive charge would have resulted in binding to the adsorbent in our specific experimental setting, as well.

As a limitation of our study, we were not able to discriminate whether the enhanced adhesion of desialylated platelets was due to desialylation as such, for example, by alteration in the platelet surface charge due to loss of negatively charged sialic acid residues, or due to platelet activation, or to both. However, recent studies have shown the influence of platelet desialylation on platelet activation and reactivity.^35,36^ In any case, alterations of sialylation patterns may occur in infection due to pathogen-derived neuraminidases or in autoimmune disease (due to antibody-mediated cleavage of sialic acids), as discussed above. Decreased platelet surface sialylation has also been described in patients suffering from acute myocardial infarction, and a recent study has demonstrated abnormal activation of lysosomal neuraminidase in coronary artery disease patients.^37^ It is well conceivable that platelets from such settings would be prone to adhesion to adsorbent polymers, as seen in our study.

## Conclusion

In conclusion, our results indicate that desialylation can contribute to enhanced interaction of platelets with adsorbent polymers during lipoprotein apheresis and may thus trigger the development of thrombocytopenia, for example, in patients with altered endogenous neuraminidase activity or in the presence of exogenous (pathogen-derived) neuraminidase.

## Supplemental Material

Supplementary_Material_revised – Supplemental material for Desialylation of platelet surface glycans enhances platelet adhesion to adsorbent polymers for lipoprotein apheresis Click here for additional data file. Supplemental material, Supplementary_Material_revised for Desialylation of platelet surface glycans enhances platelet adhesion to adsorbent polymers for lipoprotein apheresis by Lucia Lauková, René Weiss, Vladislav Semak and Viktoria Weber in The International Journal of Artificial Organs
