# Supplementary material for: Desialylation of platelet surface glycans enhances platelet adhesion to adsorbent polymers for lipoprotein apheresis
Source: Int J Artif Organs. 2020 Nov 3;44(6):378–84. doi: 10.1177/0391398820968849 (PMC8524686; doi:10.1177/0391398820968849)
Supplement: Supplementary_Material_revised – Supplemental material for Desialylation of platelet surface glycans enhances platelet adhesion to adsorbent polymers for lipoprotein apheresis [file Supplementary_Material_revised.pdf]

Supplementary Material

Desialylation of Platelet Surface Glycans Enhances Platelet Adhesion to Adsorbent Polymers for Lipoprotein Apheresis

Supplemetary Figure S1

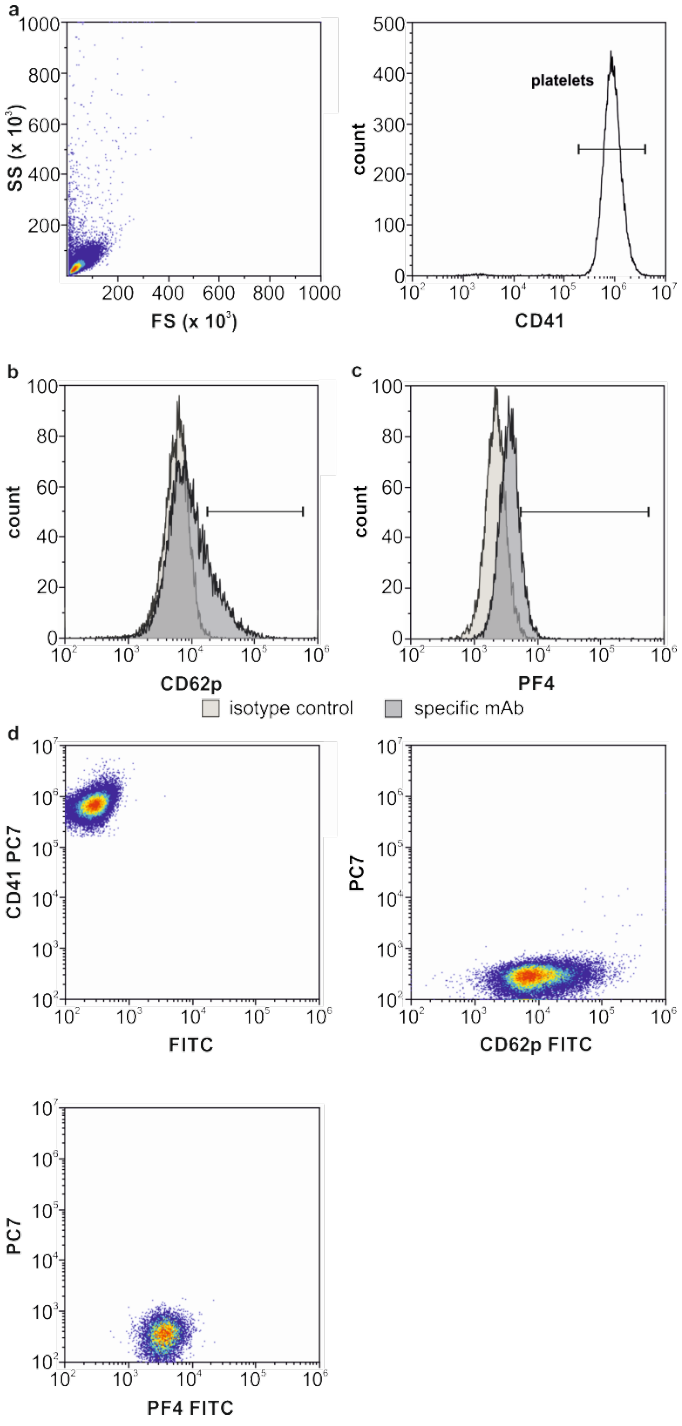

Supplementary Figure S1. Characterization of platelets and platelet activation by flow cytometry. Platelets were identified and gated based on their expression of CD41, as described in the main manuscript (panel a). P-selectin (CD62p) and platelet factor 4 (PF4) were used as markers for platelet activation. The respective isotype controls and single stainings are shown (panels b and c). Bars indicate positive expression. Flow cytometry density plots of single stainings are shown to avoid fluorescence spillover (panel d).

Supplemetary Figure S2

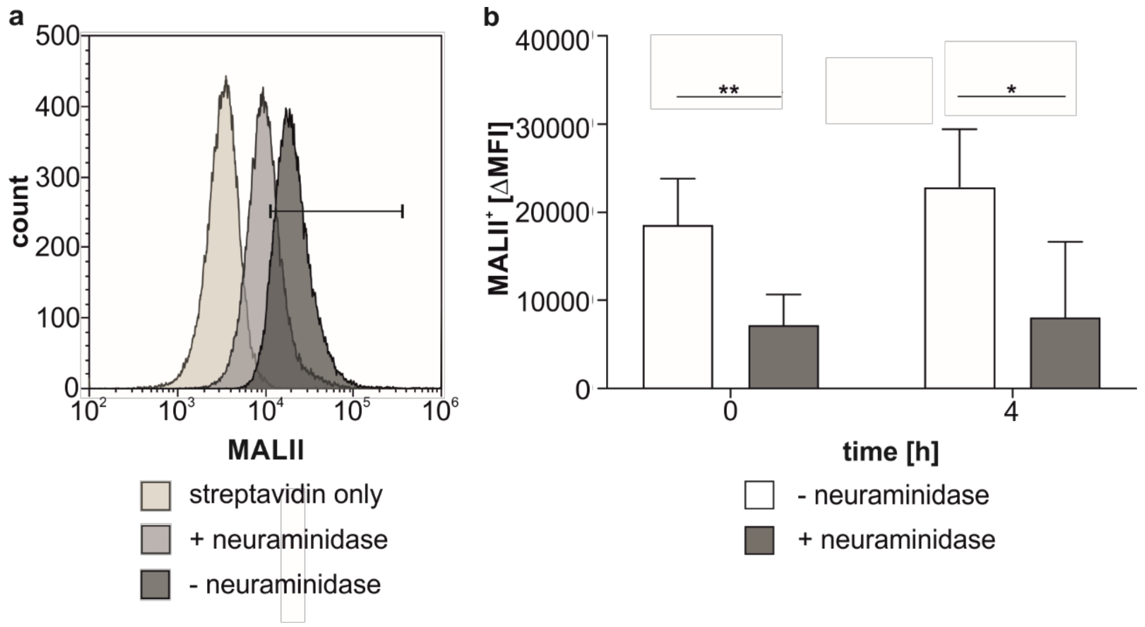

Supplementary Figure S2. Effect of neuraminidase treatment. Binding of biotinylated *Maackia amurensis* lectin II (MALII) followed by incubation with streptavidin-PE was used to detect and quantify sialic acid residues on the platelet surface after incubation with or without neuraminidase. Single staining with streptavidin-PE served as negative control (panel a). Treatment of platelet concentrates with neuraminidase as described in the main manuscript

resulted in significant reduction of terminal sialic acid residues ($MALII^+$ cells) on platelet surface glycans (panel b). **$p<0.01$, $n=4$.

Supplemetary Figure S3

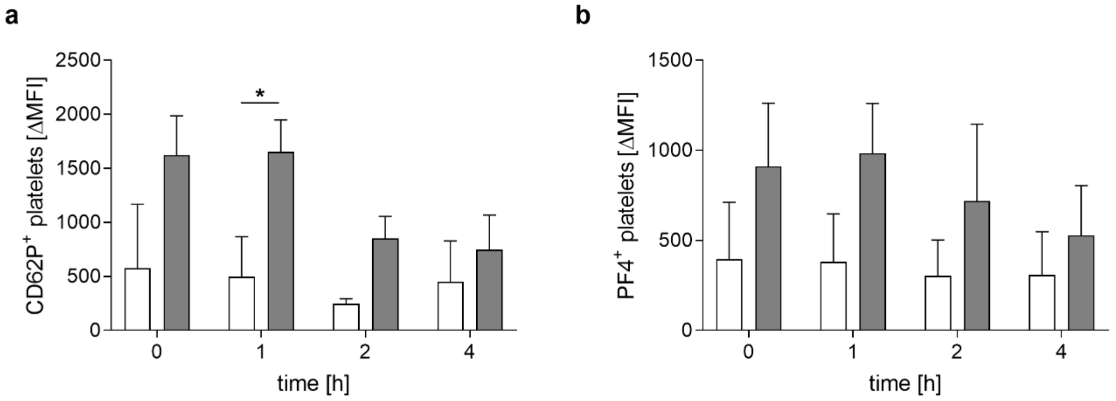

Supplemetary Figure S3. Characterization of platelet activation (ΔMFI). Platelets were treated with neuraminidase (filled bars) or were left untreated (open bars) and recirculated over columns containing polyacrylate-based DALI beads. Platelet activation was assessed by flow cytometry using P-selectin (CD62P) and platelet factor 4 (PF4) surface expressions as indicator for platelet activation (panel a and b). ΔMFI (mean fluorescence intensity) indicate the difference in MFI of the isotype control and the MFI of the specific antibody. *$p<0.05$, $n=4$.
